# Is Thrombocytopenia an In-Hospital Mortality Risk Factor among Patients with Acute Ischemic Stroke? A Propensity Score-Matched Analysis from the MIMIC-IV Database

**DOI:** 10.3390/jcm12020580

**Published:** 2023-01-11

**Authors:** Yan-Ru Wang, Li-Yu Yang, Cho-Hao Lee, Shu-Hao Chang, Po-Huang Chen, Hong-Jie Jhou

**Affiliations:** 1 Department of Internal Medicine, Tri-Service General Hospital, National Defense Medical Center, Taipei 11490, Taiwan; 2 Department of Neurology, Changhua Christian Hospital, Changhua City 50006, Taiwan; 3 Division of Hematology and Oncology Medicine, Department of Internal Medicine, Tri-Service General Hospital, National Defense Medical Center, Taipei 11490, Taiwan; 4 Department of Computer Science and Information Science, National Formosa University, Yunlin 63201, Taiwan

**Keywords:** ischemic stroke, thrombocytopenia, intensive care unit, MIMIC-IV, propensity score matching

## Abstract

(1) Background: We aimed to evaluate the aspect of thrombocytopenia in patients with acute ischemic stroke (AIS); (2) Methods: Patients with AIS were recruited in the Medical Information Mart for Intensive Care IV database from 2008 to 2019. The thrombocytopenia was defined as a platelet blood count of less than 150 K/µL. We compared the patient characteristics and clinical outcomes using propensity score matching (PSM); (3) Results: Thrombocytopenia affected 151 out of the 1236 patients (12.2%). Patients with thrombocytopenia were older (70.5 ± 12.8 vs. 68.4 ± 14.4; SMD = 0.154) and had a higher Charlson comorbidity index (7.3 ± 2.5 vs. 6.7 ± 2.7; SMD = 0.228) and acute physiology score III (44.8 ± 21.0 vs. 38.2 ± 19.1; SMD = 0.328) than those without thrombocytopenia. The risk of in-hospital mortality did not increase linearly or nonlinearly with a lower platelet count (overall p value = 0.794; nonlinear *p* value = 0.646). After PSM, 147 pairs remained. Thrombocytopenia was not linked with in-hospital mortality (HR: 1.06, 95% CIs: 0.60–1.88); (4) Conclusions: We described the clinical characteristics of patients admitted for thrombocytopenia and AIS who did not receive reperfusion therapy; additionally, we found that thrombocytopenia was not an independent short-term risk factor of in-hospital mortality.

## 1. Introduction

A stroke is defined as a neurological deficit caused by an acute focal central nervous system injury [1]. Acute ischemic stroke (AIS) accounts for 87% of all strokes, with thrombotic strokes accounting for 80% and embolic strokes accounting for 20% [2]. According to a review by Campbell et al., stroke remained the second leading cause of death and the third leading cause of disability globally [3]. Moreover, the need for intensive care for AIS has increased due to the aging population and innovative reperfusion therapy (including intravenous thrombolysis and mechanical thrombectomy). Meanwhile, subspecialized neurocritical care (NCC) delivery was linked with lower mortality and better functional results in patients with acute brain injury [4]. Hence, identifying the risk factors for poor results early on should be considered to determine the appropriate population for advanced therapy and specific NCC services.

Thrombocytopenia is a significant prognostic factor in critically ill patients. Several pathophysiological mechanisms for thrombocytopenia may contribute to this, including sepsis, inflammatory disorders, stroke, autoimmune disease, hemolytic disorders, liver impairment, resuscitation fluids causing hemodilution, or drugs like vancomycin [5,6]. Moreover, because thrombocytopenia is caused by the underlying disease, the specific treatment of the underlying disease should be considered to improve thrombocytopenia.

Thrombocytopenia is a common complication of AIS, affecting 2.3% of patients. Patients with AIS and thrombocytopenia have a higher mortality rate. Intracerebral hemorrhage (ICH) after AIS was more common in patients with thrombocytopenia [7]. Due to the risk of hemorrhaging, many trials of reperfusion therapy for AIS may exempt thrombocytopenia patients [8,9,10,11]. Moreover, platelet aggregation inhibitors and direct oral anticoagulants, which are used for the secondary prevention of ischemic stroke, may lead to a numeric increase in ICH risk without significant bleeding in addition to thrombocytopenia [12,13].

The role of thrombocytopenia in critically ill AIS patients remains unknown. Therefore, we retrospectively reviewed the propensity score matching (PSM) study on patients with AIS and thrombocytopenia using the Multiparameter Intelligent Monitoring in Intensive Care (MIMIC-IV) database to investigate the clinical characteristics and outcomes.

## 2. Materials and Methods

### 2.1. Study Population and Data Source

We enrolled patients in a single-center, retrospective cohort using the MIMIC-IV database (version: 1.0), with approval from the Institutional Review Board (IRB) of Beth Israel Deaconess Medical Center (BIDMC) and the Massachusetts Institute of Technology [14,15]. This database is an updated version from MIMIC-III, and the personal information is deidentified following the Health Insurance Portability and Accountability Act Safe Harbor provision. This large critical care database contains clinical information on patients admitted to BIDMC’s intensive care units (ICUs) between 2008 and 2019. The researcher has gained access to extract information from the database by passing a Collaborative Institutional Training Initiative examination (certification number: 39050603, Hong-Jie Jhou). Changhua Christian Hospital’s IRB approved the research protocol (IRB No. 211106).

### 2.2. Study Population and Variable Extraction

**Figure 1:**
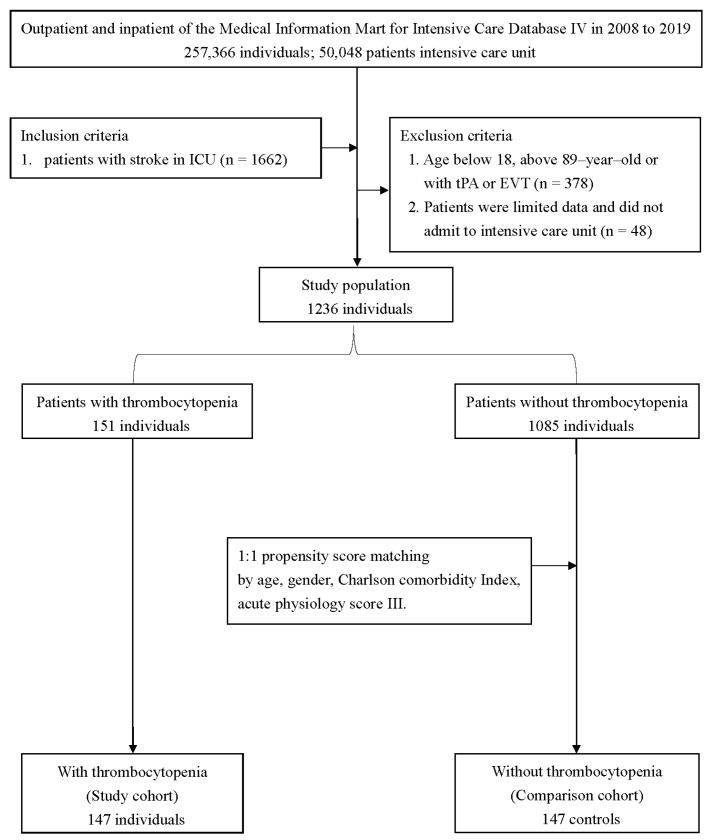
Flowchart for selecting the study samples from the Medical Information Mart for Intensive Care IV database. EVT: Endovascular thrombectomy; ICU: Intensive care unit; tPA: tissue plasminogen activator.

All patients in the MIMIC-IV database were inspected using the following criteria: (1) age greater than 18 years, and (2) ischemic stroke patients defined by ICD-9 codes of 433, 434, 436, 437.0, and 437.1 or ICD-10 codes of I63, I65, and I66 (Figure 1). We exempted patients who were receiving reperfusion therapy and patients without a platelet count. If patients were admitted to the ICU more than once, we only extracted clinical information from the first ICU admission.

Within 24 h of ICU admission, the following patient characteristics were collected: age, gender, race, diabetes, coronary artery disease, congestive heart failure, peripheral vascular disease, chronic pulmonary disease, liver disease, chronic kidney disease, malignancy, atrial fibrillation, acute physiology score (APS) III, HAS-BLED score, and the first value for vital signs and laboratory data. The Charlson comorbidity index (CCI) was calculated using 18 medical condition categories from the medical records [16,17].

### 2.3. Definition of Thrombocytopenia and Outcome Measurement

Thrombocytopenia was defined by platelet value (platelet counts of less than 150 K/µL) [18]. The primary outcome was designated as in-hospital mortality. The secondary outcomes were ICU mortality, the incidence of intracerebral hemorrhage, and percutaneous endoscopic gastrostomy, or jejunostomy tube placement.

### 2.4. Statistical Analysis

Categorical variables were conveyed as numbers with percentages, while continuous variables were presented as means with standard deviation. Cox proportional hazard models with constrained cubic splines were used to evaluate the nonlinear relationship between the platelet count and in-hospital mortality in patients with ischemic stroke. Furthermore, when an adequate cut-off value was not available, we classified the patients into two groups based on conventional definitions: thrombocytopenia and non-thrombocytopenia.

To reduce heterogeneity and selection bias, the propensity scores were calculated using the following covariates: age, gender, APS III, and CCI in a logistic regression model. Matching was performed without trimming, using a Greedy 5-to-1 Digit-Matching algorithm. In the original and PSM cohorts, univariate Cox hazards model analyses were performed to determine the relationship between thrombocytopenia and ICU and in-hospital mortality. The findings were presented as a hazard ratio (HR) with a 95% confidence interval (CI). We also ran logistic regression for the incidence of intracerebral hemorrhage before and after PSM. The results were expressed as an odds ratio with a 95% CI. For time-to-event results, the Kaplan–Meier method with a log-rank test was used to calculate the risk for in-hospital mortality between the separated groups.

All comparisons were planned, the tests were two-sided, and the statistical significance was denoted by *p* values less than 0.05. To estimate the balance of the baseline characteristics before and after PSM, a standardized mean difference (SMD) was used. An SMD less than 0.1, in general, implied a balanced characteristic between the groups. R Version 4.0.1 (R Core Team (2020), R Foundation for Statistical Computing, Vienna, Austria) was used for statistical analyses.

## 3. Results

### Main Text

**Figure 2:**
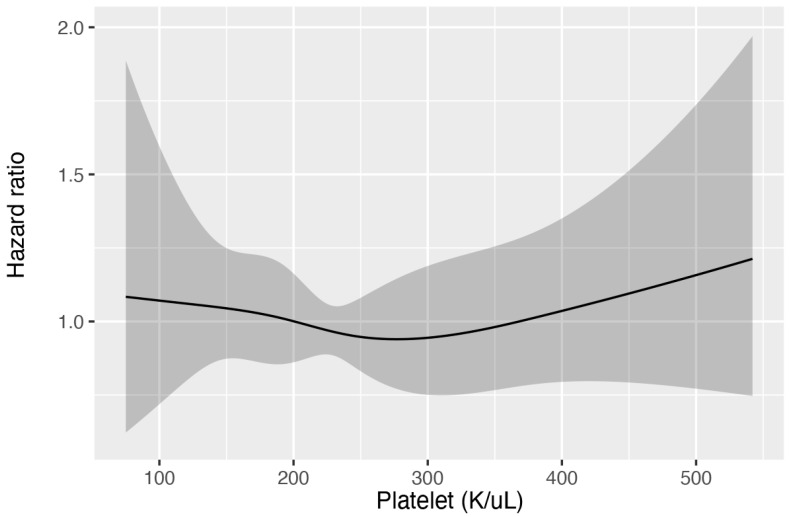
The restricted cubic splines present the nonlinear association between thrombocytopenia and in-hospital mortality. The 95% confidence interval is shown by the shaded areas around the curves.

**Figure 3:**
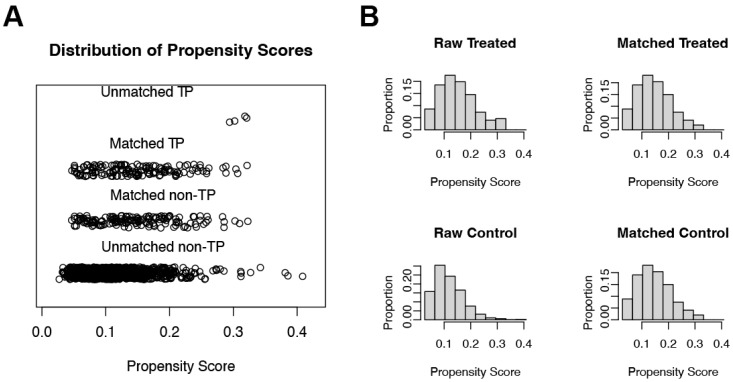
Distribution of propensity scores. (**A**) Jittered plot showing the distribution of propensity scores for the matched and unmatched subjects. An individual patient is represented by each circle. (**B**) Pre- and postmatching histograms demonstrating the density of propensity scores distribution in the TP and non-TP. TP: thrombocytopenia.

A total of 257,366 medical records were reviewed, with 50,048 patients admitted to the ICU. Among these, 1236 patients with ischemic stroke were included in the study (Figure 1). Table 1 summarizes the basic demographic characteristics. There were 151 thrombocytopenia patients and 1085 non-thrombocytopenia patients. Notably, patients with thrombocytopenia had a higher male-to-female ratio (males: 68.9% vs. 51.2%; *p* < 0.001; SMD = 0.366) and were older (70.5 ± 12.8 vs. 68.4 ± 14.4; *p* = 0.088; SMD = 0.154) than the patients without thrombocytopenia. Moreover, the patients with thrombocytopenia had a significantly higher CCI (7.3 ± 2.5 vs. 6.7 ± 2.7; *p* = 0.010; SMD = 0.228) and APS III (44.8 ± 21.0 vs. 38.2 ± 19.1; *p* < 0.001; SMD = 0.328), as well as a higher number of comorbidities, such as liver disease (3.3% vs. 0.2%; *p* < 0.001; SMD = 0.240), chronic kidney disease, malignant cancer (13.2% vs. 5.8%; *p* = 0.001; SMD = 0.255), and atrial fibrillation (45.7% vs. 34.2%; *p* = 0.006; SMD = 0.236; Table 1). Cox proportional hazard models with restricted cubic splines were used to investigate the relationship between platelet count and in-hospital mortality, and the results revealed that a lower platelet count did not increase the risk of in-hospital mortality (overall *p* value = 0.794; nonlinear *p* value = 0.646, Figure 2). We classified the patients into two groups, namely thrombocytopenia and non-thrombocytopenia, based on conventional definitions, as there was no appropriate cut-off point. The propensity scores were calculated using the following covariates: age, sex, CCI, and APS III. After 1:1 PSM, 294 patients remained in the study. Both the thrombocytopenia and non-thrombocytopenia groups were well-balanced with the four covariates (Figure 3).

Thrombocytopenia was not associated with higher ICU mortality risk (crude HR, 1.26; 95% CI, 0.60–2.61; *p* = 0.540) or in-hospital mortality (crude HR, 1.20; 95% CI, 0.69–2.10; *p* = 0.521) in the original cohort based on the univariate Cox regression analysis. Furthermore, thrombocytopenia was not associated with an increased risk of intracerebral hemorrhage (crude OR, 1.08; 95% CI, 0.55–2.12; *p* = 0.833) and PEG/PEJ tube placement (crude OR, 0.64; 95% CI, 0.30–1.37; *p* = 0.251). Notably, after PSM, all outcomes had similar outcomes (Table 2).

**Figure 4:**
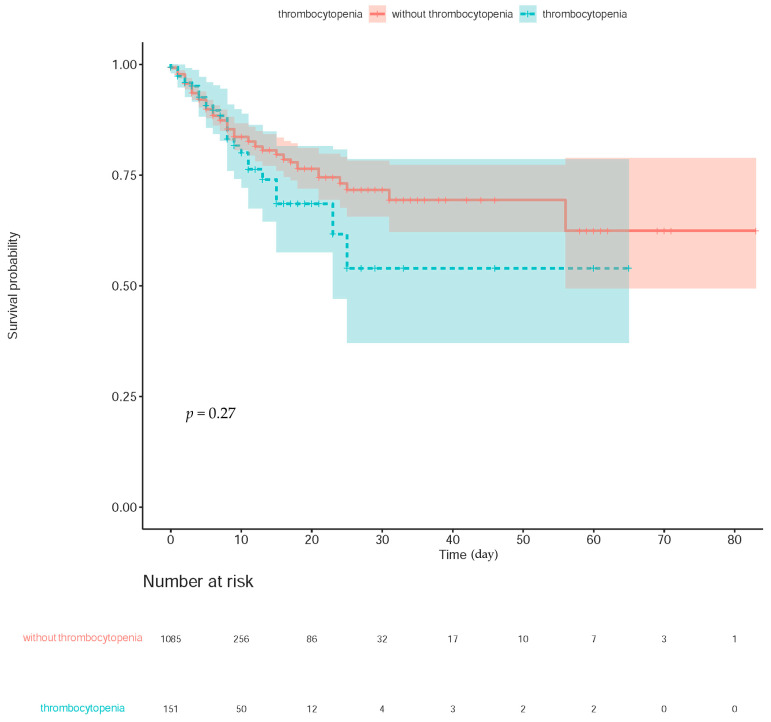
Kaplan–Meier survival curves for survival regarding in-hospital mortality. The filled color in red and green indicated the confidence interval.

The Kaplan–Meier curves revealed no link between thrombocytopenia and in-hospital mortality (Log-rank test *p* value = 0.27; Figure 4). We found that the longest length of hospital stay was 83 days, and survival was documented until discharge.

## 4. Discussion

Our retrospective study found that patients with thrombocytopenia seemed to have a higher APS III, a one-day longer ICU stay, and more comorbidities than patients without thrombocytopenia. We found that thrombocytopenia is associated with longer hospital stays and a higher risk of ICU and hospital mortality, but this relationship was not seen after PSM. Because there was no linear relationship between platelet count and in-hospital mortality, we missed the fine-cut point of platelet count for poor prognosis among critically ill patients with AIS.

Unfortunately, we could not use randomization in our observational study to reduce selection bias. Thus, the treatment and control groups possess clinical differences in the observed covariates. As a result, PSM was used to eliminate any potential bias from confounding the factors between the two groups. The propensity score, which is defined as the conditional probability of being treated given the multiple covariates, can be used to equipoise the covariates in the two groups and thus reduce these confounding factors [19]. We modified the PSM in terms of age, gender, comorbidities, and a severity scoring system to obtain an equilibrium distribution of the generalized condition, and we eliminated the heterogeneity between the compared groups. The association between thrombocytopenia and poor outcomes did not persist after PSM regarding hospital length of stay, ICU mortality, and in-hospital mortality, indicating that age, gender, comorbidities, and disease severity may be the culprits of the misleading association. However, when compared to patients without thrombocytopenia, those with thrombocytopenia spent one more day in the ICU.

Platelets are heavily involved in inflammation and immune response. Several inflammatory mediators were released by the platelets without a known role in hemostasis [20]. Platelet consumption and platelet-induced inflammation may occur as the disease progresses, and thrombocytopenia is a result of the underlying illness. As a result, we should be cautious in interpreting the link between thrombocytopenia and clinical outcomes.

According to previous research, patients with thrombocytopenia may have more pulmonary, hepatic, renal, and malignant diseases [5]. AIS is also more likely to occur in cancer patients [21]. Patients with active malignancies have poorer functional outcomes and higher mortality rates than patients without active malignancies, even after reperfusion therapy [22]. Our study also found a significantly lower white blood count in the thrombocytopenia group, which could be explained by the white blood count platelet complex hypothesis [23]. As atherosclerosis progressed, more platelet activation and atherothrombosis resulted from increased white blood cell count platelet complex formation. Therefore, the white blood cell count and platelet count may both falls at the same time.

Intracerebral hemorrhage after intravenous thrombolysis was related to higher age, higher stroke severity, and higher blood glucose level. There was approximately a tripling of the odds of intracerebral hemorrhage with the presence of atrial fibrillation, congestive heart failure, renal impairment, previous antiplatelet agents, leukoaraiosis, and a visible acute cerebral ischemic lesion on the pretreatment brain imaging [24]. Furthermore, many prognostic scores have been developed to predict intracerebral hemorrhage after intravenous thrombolysis in AIS. The Multicenter Stroke Survey includes thrombocytopenia as one of its components [25]. Low platelet counts have been linked to intracranial hemorrhage in admitted AIS patients [7] and should not be treated with endovascular thrombectomy or intravenous thrombolysis [26]. In our study, however, we found no link between TP and intracerebral hemorrhage. It may be explained that a single variable could not be used as a good prognostic factor. Prognostic scores, including multiple risk factors, may be more accurate in predicting poor clinical outcomes.

Our study has several clinical and statistical limitations. First, because this study was a retrospective study, we could not confirm diagnostic accuracy by directly evaluating patients and had to rely on diagnostic codes to define this cohort. Therefore, we might make incorrect associations via misclassifications. Second, despite the PSM and univariable analysis, the goal of correcting for clinical heterogeneity between the two groups and the potential confounding effects remains unmet owing to the nature of the MIMIC database, the potential unmeasured confounders, such as ischemic stroke subtypes (TOAST classification), National Institutes of Health Stroke Scale elimination, time of stroke, cause of thrombocytopenia, and cause of death. Moreover, we lacked discharge disposition and a modified Rankin scale at three months; hence, we could not assess the patient’s functional outcomes. Third, because patients were recruited from a single center, we addressed the issue of selection bias. Fourth, our results may not be generalizable, as our study included only critically ill patients with AIS and excluded patients receiving reperfusion therapy. Finally, the follow-up time was too short for predicting long-term outcomes.

## 5. Conclusions

Compared to patients without thrombocytopenia and AIS, those admitted for thrombocytopenia and AIS have poor clinical characteristics and prognosis. However, we did not find an accurate cut-off value for the platelet count to predict a poor prognosis. In addition, once the stroke-related comorbidities are controlled, the incorporation of thrombocytopenia may no longer be an independent risk factor.

## Figures and Tables

**Table 1:** Characteristics of the study patients.

All Patients					Propensity-Matched Pairs			
Characteristics	TP Group	Non-TP Group	*p* Value	SMD	TP Group	Non-TP Group	*p* Value	SMD
	(*n* = 151)	(*n* = 1085)			(*n* = 147)	(*n* = 147)		
Age (years)	70.5 ± 12.8	68.4 ± 14.4	0.088	0.154	70.3 ± 12.9	70.6 ± 13.7	0.833	0.025
Gender (Female), *n*	47 (31.1%)	529 (48.8%)	<0.001	0.366	47 (32.0%)	50 (34.0%)	0.710	0.043
Race, *n*			0.580 #	0.163			0.538 #	0.268
White	92 (60.9%)	728 (67.1%)			92 (62.6%)	102 (69.4%)		
Black	19 (12.6%)	98 (9.0%)			17 (11.6%)	9 (6.1%)		
Asian	5 (3.3%)	27 (2.5%)			5 (3.4%)	4 (2.7%)		
Other	35 (23.12%)	232 (21.4%)			33 (22.4%)	32 (21.8%)		
Comorbidities, *n*								
CCI	7.3 ± 2.5	6.7 ± 2.7	0.010	0.228	7.3 ± 2.6	7.3 ± 2.9	1.000	<0.001
Diabetes Mellitus								
Without chronic complication	37 (24.5%)	310 (28.6%)	0.297	0.092	37 (25.2%)	47 (32.0%)	0.197	0.151
With chronic complication	92 (8.5%)	13 (8.6%)	0.957	0.005	12 (8.2%)	10 (6.8%)	0.658	0.052
Coronary artery disease	18 (11.9%)	137 (12.6%)	0.806	0.022	18 (12.2%)	16 (10.9%)	0.715	0.043
Congestive heart failure	29 (19.2%)	196 (18.1%)	0.734	0.029	27 (18.4%)	23 (15.6%)	0.535	0.072
PVD	18 (11.9%)	129 (11.9%)	0.991	0.001	17 (11.6%)	25 (17.0%)	0.182	0.156
CPD	21 (13.9%)	198 (18.2%)	0.190	0.118	20 (13.6%)	34 (23.1%)	0.035	0.248
Liver Disease								
Mild	11 (7.3%)	26 (2.4%)	0.003 #	0.229	11 (7.5%)	4 (2.7%)	0.064	0.218
Moderate to severe	5 (3.3%)	2 (0.2%)	<0.001 #	0.240	5 (3.4%)	0 (0.0%)	0.060 #	0.265
Chronic kidney disease	37 (24.5%)	145 (13.4%)	<0.001	0.287	34 (23.1%)	21 (14.3%)	0.052	0.228
Malignancy	20 (13.2%)	63 (5.8%)	0.001	0.255	20 (13.6%)	13 (8.8%)	0.196	0.151
Atrial fibrillation	69 (45.7%)	371 (34.2%)	0.006	0.236	66 (44.9%)	60 (40.8%)	0.480	0.083
Laboratory Parameters								
WBC (10^9^/L)	9.0 ± 5.5	10.4 ± 4.4	<0.001	0.285	8.9 ± 5.5	10.8 ± 4.6	0.002	0.366
Hgb (g/dL)	12.0 ± 2.5	12.5 ± 2.1	0.010	0.209	12.0 ± 2.5	12.5 ± 2.2	0.106	0.190
Platelet (109/L)	120.2 ± 28.3	246.1 ± 87.2	<0.001	1.943	119.8 ± 28.4	237.2 ± 71.2	<0.001	2.167
Creatinine (mEq/L)	1.4 ± 1.5	1.0 ± 0.8	<0.001	0.305	1.4 ± 1.5	1.1 ± 0.5	0.013	0.292
BUN (mg/dL)	23.2 ± 15.4	18.6 ± 11.5	<0.001	0.345	23.1 ± 15.5	19.8 ± 9.2	0.027	0.260
Sodium (mmol/L)	140.0 ± 4.2	139.3 ± 3.9	0.054	0.163	140.0 ± 4.2	139.4 ± 4.9	0.219	0.144
Potassium (mmol/L)	4.1 ± 0.7	4.1 ± 0.6	0.686	0.033	4.1 ± 0.7	4.0 ± 0.6	0.226	0.141
Bilirubin (mg/dL)	0.8 ± 0.6	0.6 ± 0.5	0.001	0.361	0.8 ± 0.6	0.6 ± 0.5	0.039	0.338
INR	1.4 ± 1.2	1.2 ± 0.4	<0.001	0.207	1.4 ± 1.2	1.3 ± 0.4	0.216	0.157
HAS-BLED score	3.9 ± 1.0	3.7 ± 0.9	0.003	0.254	3.9 ± 1.0	3.7 ± 0.9	0.051	0.229
APS III	44.8 ± 21.0	38.2 ± 19.1	<0.001	0.328	43.5 ± 19.8	44.5 ± 20.2	0.671	0.050
ICU mortality, *n*	16 (10.6%)	81 (7.5%)	0.180	0.109	14 (9.5%)	14 (9.5%)	1.000	<0.001
ICU length of stay, day	5.0 ± 5.3	4.1 ± 4.9	0.035	0.179	4.9 ± 5.3	4.0 ± 4.7	0.159	0.165
In-hospital mortality, *n*	27 (17.9%)	136 (12.5%)	0.069	0.149	25 (17.0%)	22 (15.0%)	0.633	0.056
Hospital length of stay, day	8.9 ± 9.4	7.8 ± 9.2	0.169	0.118	8.6 ± 8.3	8.1 ± 9.1	0.635	0.055
Intracranial hemorrhage, *n*	17 (11.3%)	105 (9.7%)	0.542	0.052	14 (9.5%)	13 (8.8%)	0.840	0.024
PEG/PEJ tube placement, *n*	15 (9.9%)	122 (11.2%)	0.631	0.043	14 (9.5%)	19 (12.9%)	0.356	0.108

#: Tested by Fisher’s exact test. Propensity score matching by age, sex, Charlson comorbidity index, acute physiology score III, and HAS-BLED score. APS III: acute physiology score III; BUN: blood urea nitrogen; CCI: Charlson comorbidity index; CPD: chronic obstructive pulmonary disease; Hgb: hemoglobin; ICU: intensive care unit; INR: International Normalized Ratio; PEG: percutaneous endoscopic gastrostomy; PEJ: percutaneous endoscopic jejunostomy; PVD: peripheral vascular disease; SMD: standardized mean difference; TP: thrombocytopenia; WBC: white blood cell.

**Table 2:** Association between outcomes and thrombocytopenia among patients with ischemic stroke.

With Thrombocytopenia Versus without Thrombocytopenia				
Before PSM−Univariate			After PSM−Univariate	
Outcomes	Crude HR (95% CI)	*p* Value	Adjusted HR (95% CI)	*p* Value
ICU Mortality	1.26 (0.60–2.61)	0.540	0.83 (0.40–1.75)	0.629
In-hospital Mortality	1.20 (0.69–2.10)	0.521	1.06 (0.60–1.88)	0.845
Outcomes	Crude OR (95% CI)	*p* value	Adjusted OR (95% CI)	*p* value
Intracerebral Hemorrhage	1.08 (0.55–2.12)	0.833	1.09 (0.49–2.40)	0.840
PEG/PEJ tube placement	0.64 (0.30–1.37)	0.250	0.71 (0.34–1.47)	0.357

Propensity score matching by age, sex, Charlson comorbidity index, and acute physiology score III. HR: hazard ratio; ICU: intensive care unit; OR: odds ratio; PEG: percutaneous endoscopic gastrostomy; PEJ: percutaneous endoscopic jejunostomy; PSM: propensity score matching.

## Data Availability

The datasets were accessed on 1 February 2021 and are available as follows: MIMIC-IV (https://mimic.mit.edu/).

## Abbreviations

AIS	Acute ischemic stroke
APS III	Acute physiology score III; BIDMC, Beth Israel Deaconess Medical Center
BUN	Blood urea nitrogen
CCI	Charlson comorbidity index
CPD	Chronic obstructive pulmonary disease
CI	Confidence interval
EVT	Endovascular thrombectomy
Hgb	Hemoglobin
HR	Hazard ratio
ICH	Intracerebral hemorrhage
ICU	Intensive care unit
INR	International normalized ratio
MIMIC	Multiparameter Intelligent Monitoring in Intensive Care
MSS	Multicenter Stroke Survey
NIHSS	National Institute of Health Stroke Scale
NOACs	Novel oral anticoagulants
OR	Odds ratio
PSM	Propensity score matching
PEG	Percutaneous endoscopic gastrostomy
PEJ	Percutaneous endoscopic jejunostomy
PVD	Peripheral vascular disease
SIRS	Systemic inflammatory response syndrome
SMD	Standardized mean difference
SOFA	Sequential organ failure assessment
TOAST	Trial of Org10172 in Acute Stroke Treatment
TP	Thrombocytopenia
tPA	Tissue plasminogen activator
WBC	White blood cell
